# An Integrated Transcriptomic Approach to Identify Molecular Markers of Calcineurin Inhibitor Nephrotoxicity in Pediatric Kidney Transplant Recipients

**DOI:** 10.3390/ijms22115414

**Published:** 2021-05-21

**Authors:** Erika T. Rhone, Elissa Bardhi, Sai Vineela Bontha, Patrick D. Walker, Jorge A. Almenara, Catherine I. Dumur, Helen Cathro, Daniel Maluf, Valeria Mas

**Affiliations:** 1 Department of Pediatrics, Eastern Virginia Medical School, Norfolk, VA 23507, USA; rhoneET@evms.edu; 2 Surgical Sciences Division, Department of Surgery, School of Medicine, University of Maryland, Baltimore, MD 21201, USA; ebardhi@som.umaryland.edu; 3 Cardiovascular Research Center, University of Virginia, Charlottesville, VA 22908, USA; sb6ce@hscmail.mcc.virginia.edu; 4 Arkana Laboratories, Little Rock, AR 72211, USA; patrick.walker@arkanalabs.com; 5 Aurora Diagnostics–Sonic Healthcare, Bernhardt Laboratories, Jacksonville, FL 32216, USA; cdumur@auroradx.com (C.I.D.); jalmenara@auroradx.com (J.A.A.); 6 Department of Pathology, University of Virginia, Charlottesville, VA 22908, USA; hpc4f@virginia.edu; 7 Division of Transplantation, Department of Surgery, School of Medicine, University of Maryland, Baltimore, MD 21201, USA; dmaluf@som.umaryland.edu; 8 Program of Transplantation, School of Medicine, University of Maryland, Baltimore, MD 21201, USA

**Keywords:** pediatrics, kidney transplantation, calcineurin inhibitor nephrotoxicity

## Abstract

Calcineurin inhibitors are highly efficacious immunosuppressive agents used in pediatric kidney transplantation. However, calcineurin inhibitor nephrotoxicity (CNIT) has been associated with the development of chronic renal allograft dysfunction and decreased graft survival. This study evaluated 37 formalin-fixed paraffin-embedded biopsies from pediatric kidney transplant recipients using gene expression profiling. Normal allograft samples (*n* = 12) served as negative controls and were compared to biopsies exhibiting CNIT (*n* = 11). The remaining samples served as positive controls to validate CNIT marker specificity and were characterized by other common causes of graft failure such as acute rejection (*n* = 7) and interstitial fibrosis/tubular atrophy (*n* = 7). MiRNA profiles served as the platform for data integration. Oxidative phosphorylation and mitochondrial dysfunction were the top molecular pathways associated with overexpressed genes in CNIT samples. Decreased ATP synthesis was identified as a significant biological function in CNIT, while key toxicology pathways included NRF2-mediated oxidative stress response and increased permeability transition of mitochondria. An integrative analysis demonstrated a panel of 13 significant miRNAs and their 33 CNIT-specific gene targets involved with mitochondrial activity and function. We also identified a candidate panel of miRNAs/genes, which may serve as future molecular markers for CNIT diagnosis as well as potential therapeutic targets.

## 1. Introduction

Kidney transplantation (KT) remains the preferred therapy for children with end-stage renal disease [1,2,3,4]. Yet despite significant advancements in short-term outcomes [5,6,7], long-term outcomes remain suboptimal. Currently, patient survival is approximately 90% at 10 years post-transplant, but only 50–60% of allografts survive this long [7,8,9]. The mismatch between patient and graft longevity presents challenges for both pediatric patients and transplant centers, since repeat transplant not only increases the likelihood of morbidity and mortality for the child but also reduces the supply of donor organs available to other transplant candidates. Currently, 13.4% of pediatric kidney transplant waitlist candidates await a second transplant [8].

Therefore, strategies to reduce premature graft loss are of paramount importance. Late graft failure is most frequently caused by chronic renal allograft dysfunction (CRAD), which refers to the final common pathway of aggregate immunologic and non-immunologic insults resulting in the insidious loss of graft function and manifested histologically by fibrosis [10,11,12,13]. Calcineurin inhibitor nephrotoxicity (CNIT) is considered one of the primary non-immunologic factors contributing to CRAD [13,14]. In 2003, Nankivell et al. evaluated the natural history of CRAD using longitudinal protocol biopsies and found nearly universal histologic evidence of CNIT at 10 years post-KT [15]. Despite their known nephrotoxicity, calcineurin inhibitors (CNI) remain the cornerstone of maintenance immunosuppressive protocols in both adult and pediatric KT recipients [16,17].

Currently, the diagnosis of CNIT requires a kidney biopsy. However, the histologic features used to identify CNIT include permanent architectural disruption, limiting the utility of this tool to modify CNI therapy prior to the onset of irreversible damage [18,19,20,21]. Additionally, concerns regarding the specificity of CNIT histology, biopsy sampling error, and inter-observer agreement among pathologists further tarnish this gold standard and highlight the need for sensitive molecular makers of CNIT [22,23,24,25].

To date, the development of CNIT biomarkers has been hampered by an incomplete understanding of its underlying molecular mechanisms. Current reliance on late and insensitive markers such as serum creatinine precludes the identification of CNIT prior to the onset of irreversible damage. The lack of surrogate markers highlights a knowledge gap regarding the underlying molecular mechanisms of CNIT. Therefore, we sought to characterize a molecular phenotype of CNIT using an integrative approach. We hypothesized that the interplay of differentially expressed miRNAs and target mRNAs would provide the basis for a unique molecular signature for accurate pediatric CNIT diagnosis. MiRNAs, which are non-coding, endogenous single-stranded RNA that post-transcriptionally repress gene expression, have emerged as a promising class of biomarkers utilized in molecular diagnostics and have garnered interest as potential therapeutic tools [26,27]. With the utilization of miRNA and gene expression tissue profiles, the specific aim of this study is to identify (1) the critical gene pathways associated with CNIT development in pediatric KT recipients and (2) key miRNA–mRNA interactions using data integration between these two molecular layers of regulation.

## 2. Results

### 2.1. Molecular Profiling Using Microarrays

**Figure 1:**
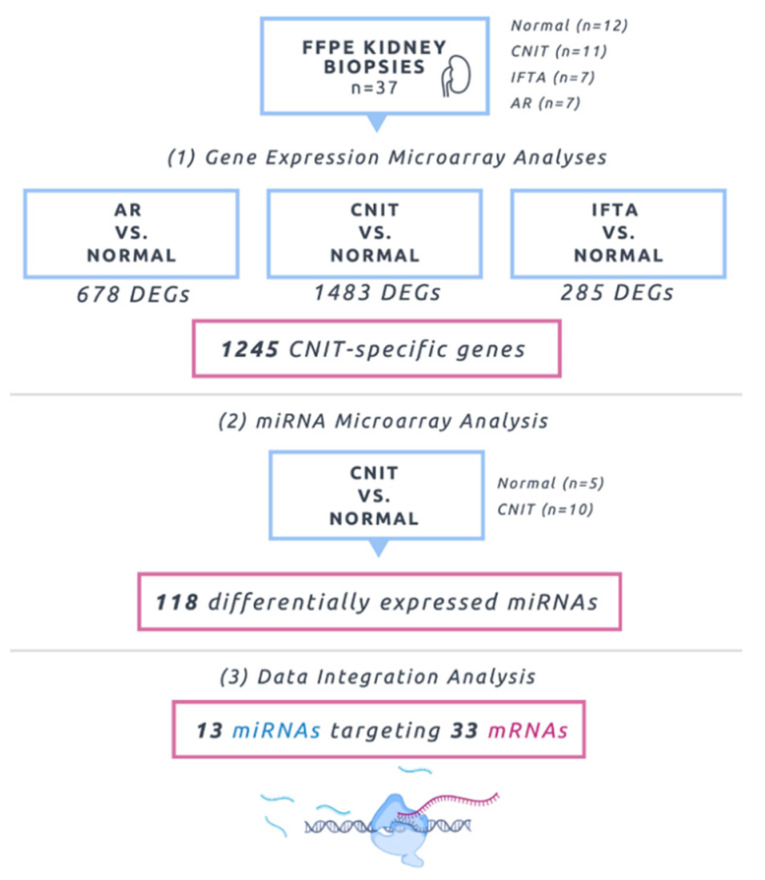
Gene expression microarrays of 37 formalin-fixed paraffin-embedded (FFPE) kidney biopsies, including Normal, calcineurin inhibitor nephrotoxicity (CNIT), interstitial fibrosis and tubular atrophy (IFTA), and acute rejection (AR), were analyzed. Then, CNIT-specific differentially expressed genes (DEGs) were integrated with 15 miRNA microarray profiles to identify miRNA–mRNA interactions.

For the gene expression analysis, 1837 probe sets were differentially expressed between CNIT and Normal samples (Table S1). This corresponded to 1483 distinct mapped genes with 714 (48%) upregulated and 769 (52%) downregulated. Two additional comparisons for marker specificity were also executed: acute rejection (AR) vs. Normal yielded 678 differentially expressed genes (60% upregulated) and interstitial fibrosis/tubular atrophy (IFTA) vs. Normal demonstrated 285 genes (31% upregulated). Figure 1 summarizes the overall study design and flow.

**Figure 2:**
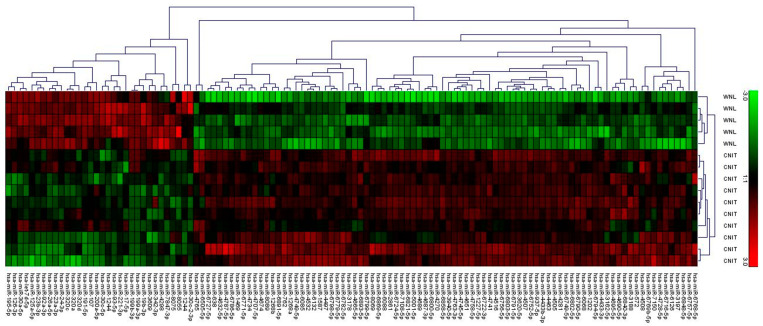
Hierarchical clustering analysis showing differentially expressed microRNAs between Normal (WNL) and CNIT allografts. MiRNA signatures in tissue samples differentiate between histological conditions.

The miRNA microarray analysis demonstrated 118 miRNAs were differentially expressed (Table S2) between Normal and CNIT samples, with a majority being upregulated (72%). A supervised hierarchical clustering was performed using miRNA expression data, demonstrating the correct grouping of CNIT and Normal cohorts (Figure 2).

### 2.2. Canonical Pathways and Upstream Regulators

The top gene pathways in the comparison between CNIT and Normal samples included oxidative phosphorylation (*p* = 3.52 × 10^−34^), mitochondrial dysfunction (*p* = 2.90 × 10^−32^), EIF2 signaling (*p* = 2.8 × 10^−16^), protein ubiquitin pathway (*p* = 2.04 × 10^−7^), and aryl hydrocarbon receptor signaling (*p* = 6.71 × 10^−7^) (Figure S1). Oxidative phosphorylation was the topmost affected pathway in this dataset, with an overlap of 54 (49.5%) out of 109 molecules and a predicted inhibition of NAD+, FAD+, and ATP (Figure S2). The 54 dysregulated genes are listed in Table S3.

**Figure 3:**
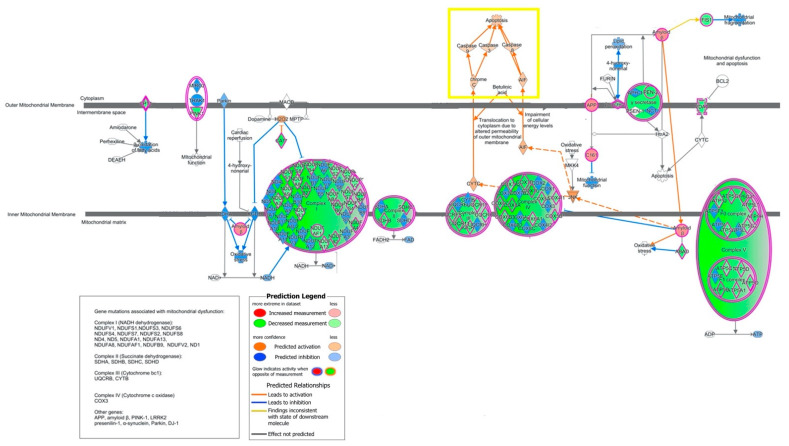
Molecule activity predictor (MAP) pathway analysis demonstrating mitochondrial dysfunction (*p* = 2.90 × 10^−32^) as one of the top pathways affected in CNIT samples. Genes associated with complexes I-V of the electron transport chain (green) were significantly downregulated. Caspases 3, 8, 9, and Cytochrome C (yellow box) are predicted to be activated, demonstrating the in-silico activation of apoptosis.

Mitochondrial dysfunction was the next most significant canonical pathway. The genes associated with complexes I-V of the electron transport chain were downregulated, with predicted inhibition of ATP production (Figure 3). There was predicted activation of Caspases 3, 8, 9, as well as cytochrome C, in its association with activation of apoptosis. Predicted directionality of EIF2 signaling indicated strong inhibition in our data (activation z-score of −3.9).

Analysis of the mostly highly significant putative upstream regulators was based upon an activation z-score and statistically significant ‘overlap *p*-values’ in IPA (*p* < 0.01). RICTOR (RPTOR independent companion of MTOR complex 2) was identified to be the top upstream regulator in the CNIT vs. Normal dataset (*p* = 4.12 × 10^−45^), with a predicted activated state (z-score 9.301). There were 97 downstream target molecules of RICTOR present in the dataset, of which 48 (49.5%) were members of the mitochondrial dysfunction/oxidative phosphorylation pathways. Additional upstream regulator analysis demonstrated a mechanistic network based on downstream gene expression patterns, which identified a connection between the activation of RICTOR and transcription factor FOXO1 inhibition. Other activated upstream regulators with the highest scores include kinases such as MAP4K4 (z-score = 5.298, *p* = 3.34 × 10^−6^) and GCK (z-score = 3.162, *p* = 2.94 × 10^−6^), the transcription regulators KDM5A (z-score = 5.298, *p* = 2.37 × 10^−10^) and PML (z-score = 2.612, *p* = 1.07 × 10^−3^), as well as mature miR-124-3p (z-score = 2.220, *p* = 1.87 × 10^−2^). VEGF was also identified as an activated top upstream regulator (z-score = 3.358, *p* = 2.85 × 10^−2^). Top upstream regulators predicted to be inhibited were comprised mainly of transcription regulators (HNF4A, z-score= −3.573, *p* = 2.23 × 10^−23^; PPARGC1A, z-score= −4.807, *p* = 5.41 × 10^−7^; TP53, z-score= −2.783, *p* = 1.34 × 10^−18^; MYCN, z-score= −4.121, *p* = 2.84 × 10^−15^).

### 2.3. Biological Characterization

The top molecular and cellular functions of the CNIT vs. Normal datasets pertained to cell death and survival (*p*-value range: 5.72 × 10^−4^–1.69 × 10^−28^), cellular growth and proliferation (p-value range: 5.52 × 10^−4^–2.66 × 10^−22^), protein synthesis (*p*-value range: 4.16 × 10^−4^–9.10 × 10^−16^), and protein degradation (*p*-value range: 1.41× 10^−4^–6.15 × 10^−16^). Functions pertaining to protein synthesis had negative activation z-scores (specifically, protein metabolism, translation, synthesis, expression). When filtered by activation z-score, free radical scavenging (specifically, the production of reactive oxygen species) was one of the highest functions (z-score = 3.263), along with cell movement (z-score = 2.593) and the differentiation of cells (z-score = 2.628). Inhibited functions were highly overexpressed in the CNIT samples related to nucleic acid metabolism and energy production (synthesis of ATP, z-score = −2.142; metabolism of nucleoside triphosphate, z-score −2.142, and synthesis of purine ribonucleotide, z-score= −2.0147).

Functional analysis with a particular focus on nephrotoxicity yielded the following results. Among the most significant renal “tox” functions included renal necrosis/cell death (p-value range: 5.98 × 10^−1^–2.23× 10^−4^, 62 dataset molecules), renal damage (*p*-value range: 2.96 × 10^−1^–4.58× 10^−3^), and renal tubule injury (p-value range 2.96 × 10^−1^–1.12 × 10^−2^). Specific functions within these categories that were especially activated included ‘apoptosis of kidney cells’ (z-score = 2.052), involving upregulation of TGFB1, TLR4, CSF1, and GFPT1 genes, in addition to ‘fibrosis of kidney’ (z-score = 2.178), with associated upregulation of TGFB1, SMAD4, TLR4, and IL2RA genes.

### 2.4. Marker Specificity, Unique Genes Associated with CNIT, and Comparison Analyses

In a comparison of IFTA vs. Normal samples, a majority of the top canonical pathways were related to metabolism: fatty acid ß-oxidation I (*p* = 1.5 × 10^−7^), TCA Cycle II (Eukaryotic) (*p* = 2.05 × 10^−4^), Tryptophan Degradation III (Eukaryotic) (*p* = 2.43 × 10^−4^), and D-glucuronate Degradation I (*p* = 5.02 × 10^−4^), in addition to mitochondrial dysfunction (*p* = 8.96 × 10^−10^). An analysis of AR vs. Normal differentially expressed genes demonstrated a predominance of immune-related canonical pathways, including Natural Killer Cell Signaling (*p* = 1.16 × 10^−18^), Th1 and Th2 Activation Pathway (*p* = 1.6 × 10^−18^), and T Cell Receptor Signaling (*p* = 2.82 × 10^−11^).

Then, a comparison analysis evaluating CNIT vs. Normal, IFTA vs. Normal, and AR vs. Normal was assessed, with a particular emphasis on canonical pathways among these three comparisons in an effort to identify distinguishing expression patterns. When classified by statistical significance, we found mitochondrial dysfunction, fatty acid ß-oxidation I, and oxidative phosphorylation to be the top pathways. Mitochondrial dysfunction and oxidative phosphorylation were the most highly enriched among CNIT vs. Normal samples (*p* = 2.92 × 10^−32^ and *p* = 3.52 × 10^−43^, respectively), while the fatty acid B-oxidation I pathway (*p* = 1.5 × 10^−7^) was predominantly expressed among IFTA samples. By activation z-score, Fc receptor-mediated phagocytosis in macrophages and monocytes, phospholipase C signaling, integrin signaling, and EIF2 signaling were all canonical pathways that were distinctly inhibited in the CNIT vs. Normal comparison.

Then, differentially expressed genes for each of the three comparisons were analyzed for overlapping (common) genes. By eliminating overlapping genes, a list of unique genes was identified for each of the conditions. A total of 66 genes were common among all three conditions. There were 418 (61.7%) unique AR genes and 107 (37.5%) unique IFTA genes. The list of 1245 (84.0%) unique CNIT genes was analyzed separately in IPA and was used for consequent data integration analyses with miRNA microarrays.

Analysis of CNIT-specific genes demonstrated a preservation of the top canonical pathways seen previously in the original CNIT vs. Normal data, including mitochondrial dysfunction, oxidative phosphorylation, and EIF2 signaling pathways. Additional gene pathways highly expressed among these CNIT genes included regulation of eIF4 and p70S6K signaling and remodeling of epithelial adherens junctions. RICTOR also remained the top upstream regulator (*p* = 1.01 × 10^−26^), while HNF4A, TP53, MYCN, and MAPT continued to be among the list of the top ten significant upstream regulators. TGFB1 and LONP1, a gene that encodes a mitochondrial matrix peptidase, were also among this list. Evaluation of the top tox lists in IPA identified mitochondrial dysfunction, renal necrosis/cell death, NRF-mediated oxidative stress response, and aryl hydrocarbon receptor signaling as overexpressed gene sets in CNIT samples.

### 2.5. Integrative Analysis (miRNA–mRNA Interactions)

**Figure 4:**
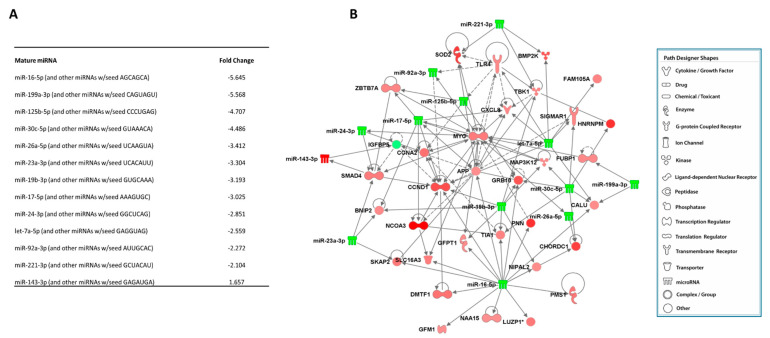
(**A**) List of 13 differentially expressed miRNAs in CNIT samples. (**B**) Integrated network of 13 miRNAs and their downstream up- (red) and down- (green) regulated genes.

Data integration was performed using unique CNIT genes and differentially expressed miRNAs between CNIT and Normal groups. This generated a filtered list (experimentally observed and with appropriate expression directionality) of 13 miRNAs and their 33 mRNA targets. An integrated network of each of these 13 miRNAs and their downstream up- and down-regulated genes are represented in Figure 4.

**Figure 5:**
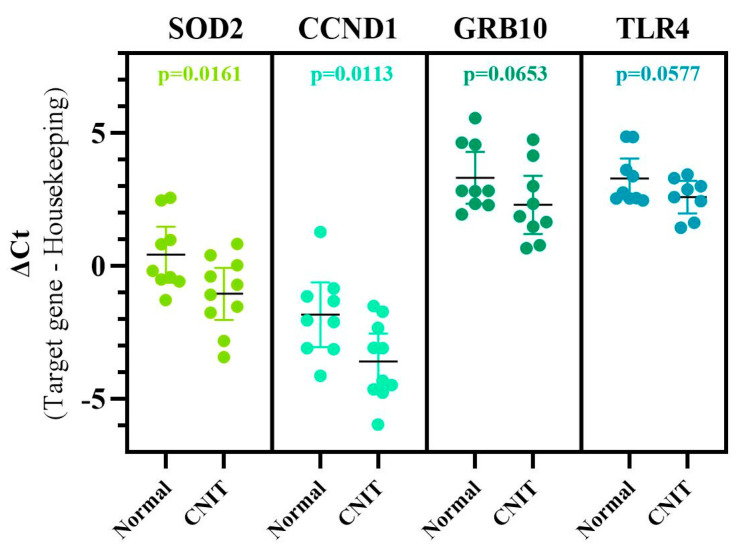
QPCR validation of four candidate genes using CNIT and Normal samples. Plotted with mean values and 95% confidence intervals. Gene expression normalized using the GAPDH housekeeping gene. A decrease in ΔCt represents an increase in gene expression. SOD2 and CCND1 were significantly upregulated, while GRB10 and TLR4 showed a trend in significance.

Then, these 13 miRNAs and 33 gene targets were further analyzed for biologic significance. First, the genes were examined for their role in canonical pathways. SOD2 and APP were genes participating in mitochondrial dysfunction, while AGO4, CCND1, and MYC were identified as being involved with EIF2 signaling. The SOD2 gene is also included in superoxide radical degradation and NRF2-mediated oxidative stress response pathways. Then, specific tox lists were evaluated for both miRNA:mRNA pairs, with SOD2, APP, CCND1, miR-16-5p, and MYC being associated with the decreased transmembrane potential of mitochondria. APP was additionally identified as a gene related to increasing the permeability transition of the mitochondrial membrane. With particular attention to nephrotoxicity tox lists, APP, GFPT1, GRB10, MYC, SOD2, and TLR4 were identified as genes related to renal necrosis/cell death and apoptosis. Based on a functional analysis for nephrotoxicity, the TLR4 gene was associated with ischemia–reperfusion injury and tubular injury, while GFPT1, TLR4, GFPT1, and SMAD4 genes were identified to be involved with glomerular injury. Other functions included renal fibrosis (SMAD4), renal cell proliferation (PNN), renal cell viability (APP), and hyperplasia (MYC). Using QPCR, we validated the increase in expression of SOD2 (*p* = 0.0161), CCND1 (*p* = 0.0113), GRB10 (*p* = 0.0653), and TLR4 (*p* = 0.0577) in CNIT samples when compared to Normal (Figure 5). GRB10 and TLR4 were not statistically significant, although a trend in significance was observed. This is likely due to the relatively small sample size and supports the need of larger studies for further validation of findings.

## 3. Discussion

This study presents a novel integrative approach to evaluate the molecular signature of CNIT in pediatric KT recipients. This study is the first to examine tissue profiles of CNIT in an exclusively pediatric KT cohort. CNIT is of particular clinical significance given its contributions to CRAD and associations with graft longevity.

Previously, our group reported the molecular profile of CNIT in adult KT recipients [28]. There is reason to believe that age-related and developmental differences in host immune system responses, drug handling (both pharmacokinetics and pharmacodynamics), primary kidney disease, and presence of concurrent cardiovascular co-morbidities may impact gene expression [29,30,31,32,33,34,35,36]. For instance, KT recipients less than 5–6 years of age often require higher CNI doses than older patients, which were potentially related to CYP3A maturation and activity [37,38,39,40]. Other reports have demonstrated more intense immune responsiveness among pediatric recipients, suggesting that the immunological milieu for the allograft may be affected by age [36,41,42]. Furthermore, children are prioritized to receive deceased donor kidneys with lower kidney donor profile index (KDPI) scores (<35%), and thus, the quality of the graft may influence gene expression [8]. Furthermore, the need for sensitive markers of allograft injury may be greater among children given that adult-sized grafts may mask damage that is not accompanied by an increase in serum creatinine [41]. While most biomarker research initially occurs in adult populations and then is extrapolated to children, this study demonstrates the value of primary pediatric biomarker discovery in transplantation.

The focus of this study was to identify molecular pathways associated with CNIT among pediatric KT recipients. We found mitochondrial dysfunction and oxidative phosphorylation to be the two central gene pathways in kidneys with CNIT, even following the elimination of commonly differentially expressed genes among IFTA and AR. Specifically, ATP depletion, increased membrane permeability, and the production of reactive oxygen species emerged as key biological processes enriched in CNIT samples. Based on in silico modeling, inhibition of genes involved in complexes I–IV of the electron transport chain predicted the downstream effect of apoptosis activation via caspases 3, 8, and 9 and cytochrome C. EIF2 signaling was also robustly inhibited in CNIT-specific genes. Among the functions of the EIF2 pathway is to serve as an mRNA translational checkpoint as part of an integrated cellular stress response [43]. Upon exposure to environmental stress, the phosphorylation of EIF2 precludes the formation of the 43S pre-initiation complex required for translation, which is a homeostatic mechanism to down-regulate protein synthesis in cellular injury [44]. Here, the inhibited expression pattern of EIF2 suggests a global reduction in protein synthesis.

Our upstream regulator analysis identified RICTOR as the top activated regulator among CNIT-specific genes. RICTOR, as a subunit of MTORC2, has been shown to have a significant role in actin cytoskeleton organization [45,46,47]. Recently, RICTOR has been implicated in renal fibrosis development, specifically in association with TGFB1-induced fibroblast activation and epithelial–mesenchymal transition [48,49]. Previous studies have highlighted the biologic connection between RICTOR/MTORC2 and mitochondrial activity [50]. MTORC2 has been localized to both endoplasmic reticulum and mitochondria, but it has a functional relationship to mitochondria through Akt phosphorylation [50,51,52]. The RICTOR/MTORC2 complex phosphorylates Akt, a serine/threonine kinase involved in cell death and cell cycle progression [52,53,54]. Both the inhibition and activation of Akt have been associated with apoptosis and increased vulnerability to oxidative stress [55,56]. Our results demonstrate that RICTOR may be an important upstream regulator involved in the cross-talk among these pathways, with roles in both mitochondrial dysfunction and fibrosis development.

Although it was not our primary objective to characterize the molecular profiles of AR and IFTA samples, we did observe distinct gene expression patterns within these groups. Unsurprisingly, the principal gene pathways in AR were related to immunity and inflammation, including activation of Th1/Th2 alloresponse, iCOS signaling in T Helper Cells, and NK cell signaling. Predicted significant upstream regulators were cytokines (IL-2, IL-15, and interferon-gamma). IFTA samples were distinguished by gene pathways and molecular functions associated with decreased metabolism (specifically, lipid metabolism) and energy production, which is largely in concordance with previous reports [56,57,58].

There has been an emerging focus on mitochondria as key arbitrators of both acute kidney injury and chronic kidney diseases [59,60,61]. With regard to CNIT, the association between mitochondrial dysfunction and CNIs has been previously described by others [18,62,63,64,65,66,67,68]. While it remains unknown whether the observed mitochondrial dysfunction is secondary to endothelial damage associated with ischemia (i.e., CNI vasculopathy) or direct tubular toxicity, the findings of our study support the assertion that mitochondrial oxidative phosphorylation defects play a role as one of the central mechanisms of CNIT development. Given that the proximal tubular epithelium is mitochondria-rich and highly ATP-dependent, these cells may be particularly damaged as a result of CNIT [69,70,71]. However, oxidative stress has also been linked with endothelial dysfunction in CNIT, with a recent paper highlighting the role of TLR4 signaling and induction of vascular inflammation (TLR4 gene up-regulated in our data) [72].

Previous investigations of mitochondria and CNIT have utilized animal or in vitro models, making this among the first studies to demonstrate mitochondrial dysfunction in association with CNIT by evaluating gene pathways in human tissue. Our focus on canonical pathways permits the study of expression patterns of genes of interest within those pathways. Furthermore, our approach allows for the identification of important mRNA–miRNA interactions in association with these gene pathways and biological functions, incorporating yet another -omics layer to further delineate the molecular mechanisms of CNIT. Of the 13 miRNAs identified in the integrative network, several have been cited in the literature as being regulators of mitochondrial activity, including miR-16-5p, the miR-30 family, miR-26, and miR-24 [73,74,75,76].

The findings in this study are strengthened by the systems biology holistic approach. Systems biology is an essential schema for the evaluation of interrelated networks derived from multi-omic data representing multiple layers of genomic regulation [77,78]. Such a framework is necessary for understanding the KT model, given the complex and dynamic interactions between recipient host and donor allograft. This study investigated the interaction between two layers of –omic regulation (miRNAs and their mRNA targets in the same samples) to further understand the molecular underpinnings of the CNIT transcriptome. Our secondary objective was to identify a panel of candidate miRNA–mRNA targets among CNIT-specific genes to provide the basis to facilitate diagnostic biomarker discovery and prospective study. A biomarker panel comprised of miRNAs holds additional promise as a therapeutic target by utilizing antagomirs as drugs to suppress miRNA expression [79,80]. Therefore, identifying key miRNAs in CNIT may represent a path toward drug development in ameliorating its nephrotoxicity.

It is important to note that this pilot study does have the additional strength of inclusion of archival tissue samples. Formalin-fixed paraffin-embedded (FFPE) tissue samples are readily available, pose no additional risk to patients, and mitigate the challenge of obtaining adequate fresh tissue specimens for study. Additionally, archived FFPE tissue blocks represent a vast resource given its existence as part of highly annotated repositories/tissue banks. The amount of tissue used as RNA input was minimal (two to three 10-micron sections), preventing the risk of “exhausting” the sample and allowing use of small biopsy cores. Over the past several years, archived FFPE samples have emerged as a suitable tissue source for various -omics platforms as a result of advances in RNA and DNA isolation technology [81,82,83,84]. This study demonstrates the successful utilization of FFPE samples as an RNA source for both gene expression and miRNA microarrays.

The limitations of the study include its small sample size and cross-sectional study design. Nonetheless, our study is one of the first of its kind to be performed in an exclusively pediatric KT cohort, with the use of only well-characterized biopsy samples (i.e., only samples without mixed diagnoses were utilized). The reported findings are based on a cross-sectional examination of transcriptomic and epigenetic changes associated with pediatric CNIT, and they are intended to serve as a framework for future studies that will improve the knowledge of pertinent molecular pathways. An essential next step will be to validate the identified panel of markers in a large, prospective pediatric cohort with protocol biopsies and well-defined clinical and demographic characteristics.

## 4. Materials and Methods

### 4.1. Study Cohort

Thirty-eight FFPE kidney biopsies from pediatric KT recipients were available for study, 12 of which represented cases with CNIT. Normal allografts served as negative controls (*n* = 12), while 14 samples made up the positive control group, which functioned to determine marker/pathway specificity, including AR group (*n* = 7) and IFTA group (*n* = 7). The AR group included 5 patients with acute cellular rejection (ACR) and 2 patients with antibody-mediated rejection (AMR).

Of the 38 biopsy samples utilized for gene expression, 1 CNIT sample did not pass hybridization quality control with a %P of < 50%. The final dataset for gene expression was comprised of 37 total samples (11 CNIT; 12 Normal; 7 AR; 7 IFTA). All 15 miRNA microarrays were utilized for analysis.

No subjects were recruited as part of this study, and only de-identified archival FFPE tissue blocks were utilized. This study was verified by the Institutional Review Board at the University of Virginia (IRB-HSR # 18482) to meet the criteria for review exemption. Inclusion criteria for participation in this study include patients ≤18 years of age with biopsies taken over 6 months post-transplantation. Normal allograft protocol biopsies were taken 12 months post-transplantation.

CNIT was defined by the histological features of isometric vacuolization of the proximal convoluted tubules, nodular hyalinization of arterioles and small arteries, or striped interstitial fibrosis—in the absence of rejection, acute tubular necrosis, and/or IFTA [20,85]. ACR, AMR, and IFTA were defined as specified by the Banff criteria [85,86,87].

### 4.2. RNA Isolation and Microarray Hybridization

Total RNA was isolated (High Pure RNA Paraffin Kit, Roche, IN, USA) per manufacturer instructions. For each sample, three 10-micron sections (de-paraffinized in xylene) were later pooled in order to increase RNA yield. Assessment of RNA concentration and purity was performed using spectrophotometry (NanoDrop ^®^ 1000). Only those samples demonstrating (1) enough quantity of RNA (>200 ng of RNA input) and (2) high-quality RNA (ratios of absorbance at 260 nm to 280 nm between 1.9 and 2.1) were considered suitable for downstream reactions. RNA was amplified and labeled (SensationPlus™ Kit; Affymetrix, Santa Clara, CA, USA), and consequently hybridized to miRNA and gene expression microarrays (Affymetrix™GeneChip HG-U133A 2.0 microarray, GeneChip miRNA v4.0 array, Santa Clara, CA, USA). Arrays were scanned with a GeneChip™ Scanner 3000 (GEO accession number GSE174020).

### 4.3. Gene Expression/miRNA Microarrays

All 38 biopsies were evaluated using gene expression microarrays. A subset of 15 biopsies was used for miRNA evaluation (*n* = 10 CNIT, *n* = 5 Normal). In order to further refine the molecular signature for CNIT, AR and IFTA samples were included for marker specificity as a “clean-up technique” to identify specific genes and pathways uniquely associated with CNIT and exclude common genes of allograft injury. The AR group was a composite of both ACR and AMR as representative of immune-mediated injury. IFTA was included as an archetype for chronic renal injury.

### 4.4. Gene Expression/miRNA Microarray Analysis and Quality Control

Raw intensities for each probeset were stored as electronic files (.CEL format). The robust multiarray average (RMA) method was used for background correction, which was followed by quantile normalization in the R environment [88,89,90,91]. A probeset level *t*-test was used to compare conditions. A *p*-value < 0.001 (<0.005 for miRNA microarrays) and q-value probeset-specific false discovery rate (FDR) ≤ 5% (Benjamini and Hochberg method [92,93]) were utilized to identify differentially expressed genes and miRNAs, respectively. Fold-change (genes ≥ 1.5 fold change and miRNAs ≥ 2) was used for relative quantification of gene and miRNA expression differences between groups. Only microarrays that passed quality control using intensity values of RNA spike-in controls were included.

Given the high likelihood of degraded RNA present in FFPE tissue, stringent hybridization quality control measures were applied [94]. The percentage of probesets declared present (%P) by the detection call algorithm: (cut-off > 50% ± 15%) and 3′:5′ ratios of the intensity values for glyceraldehyde-3-phosphate dehydrogenase (GAPDH) housekeeping gene (cut-off: ≤3).

### 4.5. Interaction Networks, Functional/Pathway Analysis, Upstream Regulators

Ingenuity Pathway Analysis™ (IPA, http://ingenuity.com, accessed 10 May 2021) software was used to identify experimentally verified and high confidence target gene interactions to define miRNA:mRNA pairs for input into pathway enrichment analysis. MiRNA and mRNA datasets were also integrated and subjected to network analyses by IPA to identify plausible associations and potential regulatory networks relating to the progression of CNIT. Specifically, gene ontology, disease relevance, and functional and network analyses were assessed. Predicted upstream regulators with significant overlap *p*-value (defined as the *p*-value associated with the degree of overlap between observed genes present in the dataset and predicted genes of an upstream regulator) were also identified. Separate analyses were performed for CNIT vs. Normal, IFTA vs. Normal, and AR vs. Normal. Then, a comparison analysis of the three conditions was evaluated, where overlapping differentially expressed genes represented common genes, while non-overlapping genes were considered unique. Statistical significance associated with networks, pathways, and functions was calculated in IPA using a Fisher’s exact test, with *p*-values < 0.05 considered significant. Predictions regarding the activation state of a function, molecule, or pathway were inferred using the IPA regulation z-score algorithm (positive and negative z-scores predict activation and inhibition respectively), which is computed based upon the observed directionality gene expression changes present in the dataset.

### 4.6. Data Integration Analysis

A separate analysis was performed using unique genes for CNIT (those remaining following the elimination of common genes expressed by IFTA and AR vs. Normal samples). Then, an integration step was carried out between differentially expressed miRNA (CNIT vs. Normal) and this dataset of unique CNIT genes (IPA microRNA Target Filter tool, which is based on content from TarBase, TargetScan, miRecords, and published literature). This analysis generated a signature of differentially expressed miRNAs and their target mRNAs present in both datasets. The final miRNA:mRNA pairs represented a filtered list of only those experimentally observed relationships pairing in the proper directions (upregulated miRNAs and their downregulated gene targets and vice versa).

## 5. Conclusions

Our results have identified mitochondrial dysfunction and oxidative phosphorylation defects to be central gene pathways involved in the pathogenesis of CNIT, with renal cell death/necrosis and energy failure/ATP depletion as salient biological functions. Additionally, we discovered a panel of miRNAs and their gene targets representing a candidate molecular signature of CNIT in pediatric KT recipients. Delineation of the molecular pathways specific for CNIT offers mechanistic insights into a clinically significant problem and is a necessary first step for the development of minimally invasive biomarkers, which may allow individualized immunosuppressive therapy and improve graft survival and quality of life for children.

## Data Availability

Arrays were scanned with a GeneChip™ Scanner 3000 (GSE174020).

## Supplementary Materials

The following are available online at https://www.mdpi.com/article/10.3390/ijms22115414/s1. Figure S1: Top 10 canonical pathways of CNIT. Figure S2: Expected vs. observed oxidative phosphorylation pathway. Table S1: Differentially expressed probesets between CNIT and Normal samples (provided separately as Excel Sheet). Table S2: Differentially expressed CNIT-specific genes related to oxidative phosphorylation. Table S3: Differentially expressed miRNAs between CNIT and Normal samples.
