# Supplementary material for: An Integrated Transcriptomic Approach to Identify Molecular Markers of Calcineurin Inhibitor Nephrotoxicity in Pediatric Kidney Transplant Recipients
Source: Int J Mol Sci. 2021 May 21;22(11):5414. doi: 10.3390/ijms22115414 (PMC8196602; doi:10.3390/ijms22115414)
Supplement: Supplementary file 1 [file ijms-22-05414-s001.zip › ijms-1199334-supplementary/CNIT supplementary files/Supplementary materials CNIT 5-4-21.pdf]

Supplemental Figure S1. Top ten significant canonical pathways of CNIT-specific core analysis using IPA. Pathways are presented in descending order of statistical significance. Genes with negative z-scores are represented in green and positive z-scores in red. The percent of CNIT-specific genes altered within each pathway is shown by the top x-axis, with the total number of genes pathway displayed at the end of each horizontal row.

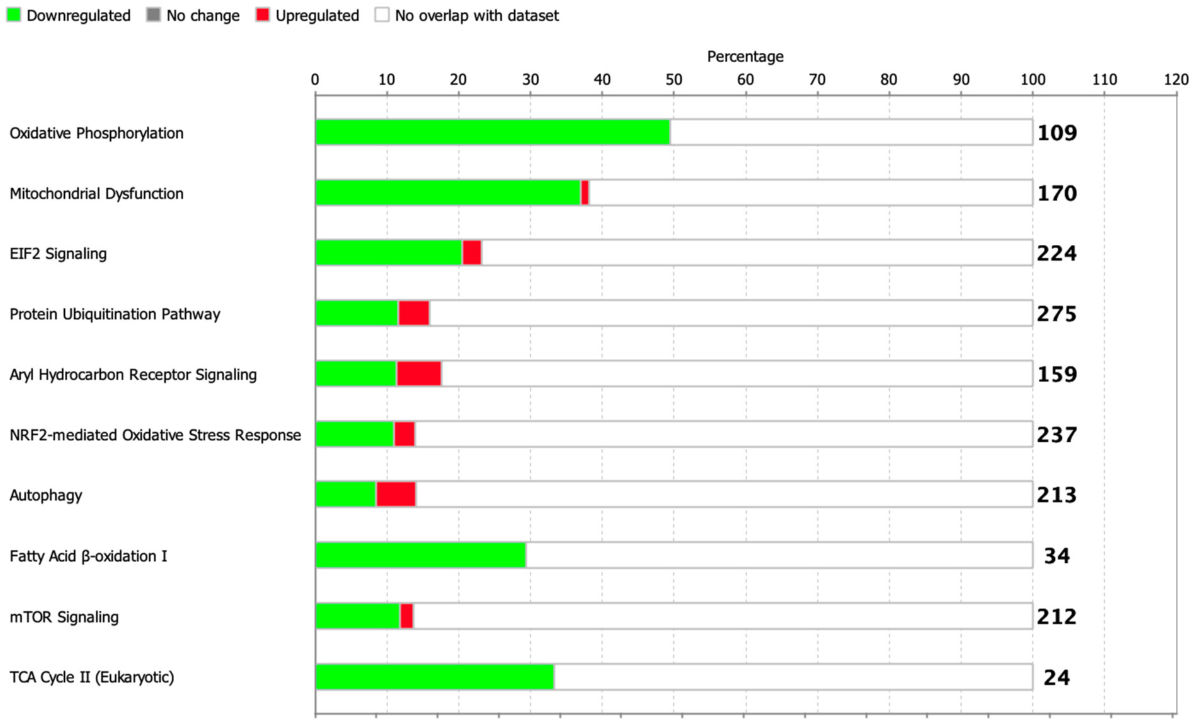

Supplemental Figure S2. A. Expected activation state of oxidative phosphorylation from the IPA knowledge database. **B.** Observed measurements of genes involved in oxidative phosphorylation in CNIT samples and predicted downstream inhibition (blue) of NAD+, FAD+, and ATP.

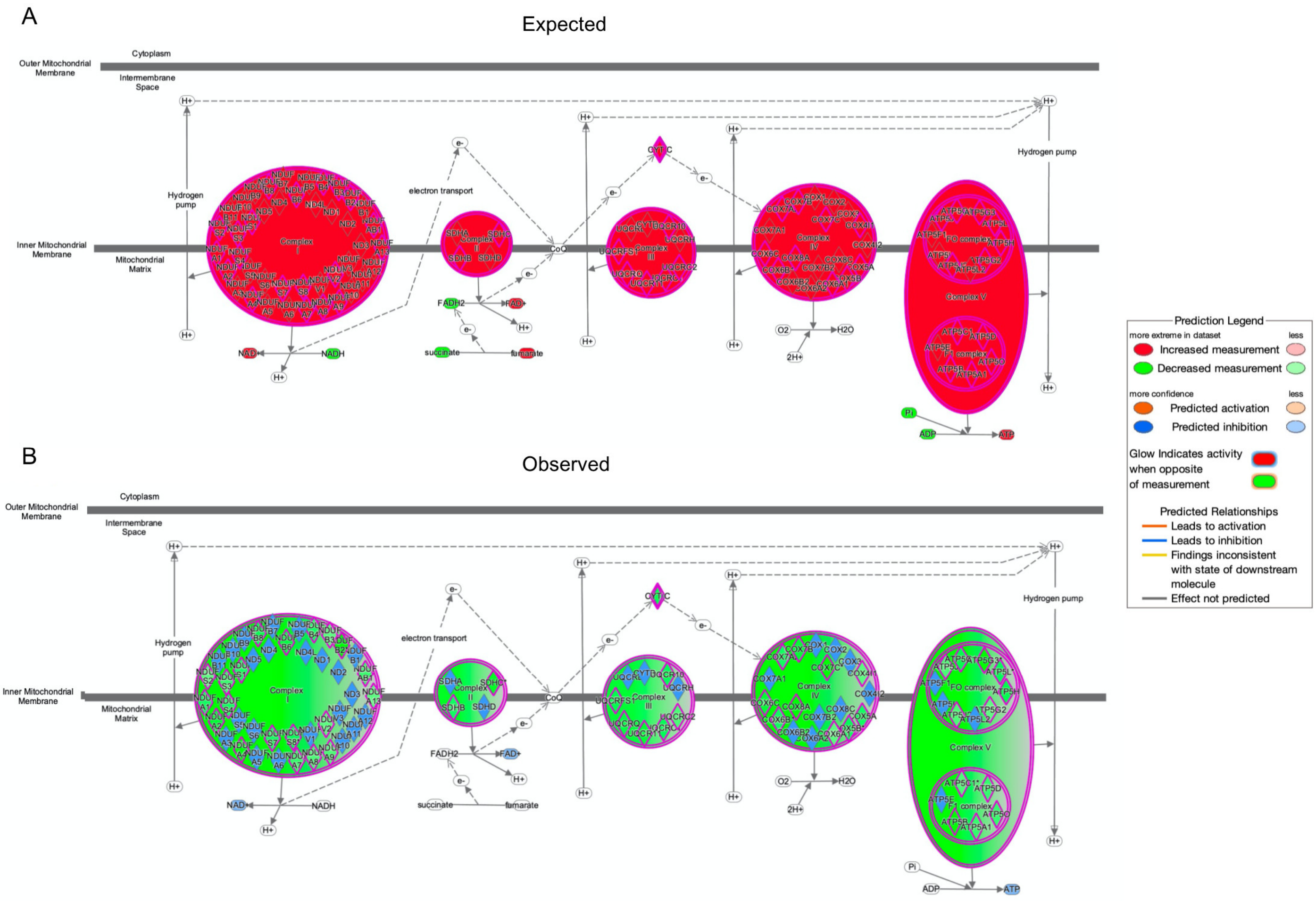

Supplementary Table S1. Differentially expressed genes between CNIT and Normal samples (FDR <5%, FC≥1.5)

[Provided separately as Excel Sheet]

Supplemental Table S2. Differentially expressed microRNAs between CNIT and NA samples (FDR <5%).

ProbeSet	Transcript ID	Fold Change	p-value (a-level= 0.005)	q-value (FDR= 5%)
20523000	hsa-miR-6068	7.8	2.52E-06	3.43E-04
20525541	hsa-miR-6790-5p	5.5	6.21E-05	3.13E-03
20506795	hsa-miR-1202	4.5	2.72E-06	3.46E-04
20504295	hsa-miR-572	4.1	3.80E-04	1.16E-02
20518940	hsa-miR-1587	4.1	8.14E-06	6.64E-04
20506006	hsa-miR-937-5p	3.7	1.09E-07	4.53E-05
20518904	hsa-miR-4507	3.5	1.11E-07	4.53E-05
20519497	hsa-miR-4689	3.4	1.84E-04	7.02E-03
20525565	hsa-miR-6802-5p	3.3	1.77E-05	1.29E-03
20524053	hsa-miR-6132	3.3	2.09E-05	1.33E-03
20525657	hsa-miR-6848-5p	3.2	1.60E-03	3.47E-02
20525383	hsa-miR-6716-5p	3.1	2.81E-04	1.01E-02
20518901	hsa-miR-4505	3.1	8.77E-09	1.79E-05
20519498	hsa-miR-4690-5p	3.0	3.43E-04	1.09E-02
20529799	hsa-miR-8089	3.0	1.63E-04	6.50E-03
20525747	hsa-miR-6893-5p	2.9	3.27E-04	1.06E-02
20519592	hsa-miR-4741	2.9	5.84E-06	4.96E-04
20518845	hsa-miR-4463	2.8	3.19E-08	3.25E-05
20517833	hsa-miR-3620-5p	2.8	4.01E-07	1.02E-04
20519457	hsa-miR-4665-5p	2.8	1.45E-04	6.16E-03
20518933	hsa-miR-4532	2.7	9.43E-05	4.37E-03
20519433	hsa-miR-4651	2.7	1.34E-06	1.96E-04
20519626	hsa-miR-4758-5p	2.7	3.72E-07	1.02E-04
20520198	hsa-miR-5001-5p	2.7	6.76E-05	3.28E-03
20518807	hsa-miR-4433-3p	2.6	1.20E-04	5.44E-03
20525603	hsa-miR-6821-5p	2.6	2.33E-05	1.40E-03
20515624	hsa-miR-3188	2.6	1.71E-03	3.55E-02
20515641	hsa-miR-3197	2.6	1.63E-03	3.47E-02
20519600	hsa-miR-4745-5p	2.6	9.74E-06	7.64E-04
20515585	hsa-miR-3162-5p	2.6	1.58E-03	3.47E-02
20525459	hsa-miR-6749-5p	2.6	1.88E-05	1.32E-03
20523017	hsa-miR-6085	2.5	5.74E-06	4.96E-04
20525465	hsa-miR-6752-5p	2.4	5.99E-07	1.36E-04
20525394	hsa-miR-6722-3p	2.4	4.89E-06	4.54E-04
20506771	hsa-miR-1227-5p	2.4	3.17E-06	3.73E-04
20529131	hsa-miR-4433b-3p	2.4	1.89E-07	6.42E-05
20519607	hsa-miR-4749-5p	2.4	1.23E-04	5.44E-03
20525519	hsa-miR-6779-5p	2.4	9.54E-07	1.77E-04

20525559	hsa-miR-6799-5p	2.4	1.11E-03	2.60E-02
20524036	hsa-miR-6126	2.4	7.02E-05	3.33E-03
20525561	hsa-miR-6800-5p	2.3	8.73E-04	2.10E-02
20525557	hsa-miR-6798-5p	2.3	9.28E-07	1.77E-04
20517736	hsa-miR-4281	2.3	3.47E-06	3.73E-04
20525549	hsa-miR-6794-5p	2.3	4.72E-05	2.53E-03
20525497	hsa-miR-6768-5p	2.3	2.30E-03	4.30E-02
20525525	hsa-miR-6782-5p	2.3	2.04E-05	1.33E-03
20519636	hsa-miR-4763-3p	2.3	4.87E-06	4.54E-04
20525543	hsa-miR-6791-5p	2.3	1.06E-07	4.53E-05
20525503	hsa-miR-6771-5p	2.2	6.30E-05	3.13E-03
20515607	hsa-miR-3178	2.2	7.24E-04	1.87E-02
20518892	hsa-miR-4497	2.2	3.39E-06	3.73E-04
20519570	hsa-miR-4728-5p	2.2	4.12E-04	1.24E-02
20525473	hsa-miR-6756-5p	2.2	1.94E-05	1.32E-03
20519405	hsa-miR-4632-5p	2.2	4.57E-04	1.30E-02
20525567	hsa-miR-6803-5p	2.2	4.08E-06	4.16E-04
20525743	hsa-miR-6891-5p	2.1	5.46E-04	1.49E-02
20525475	hsa-miR-6757-5p	2.1	6.37E-04	1.68E-02
20506865	hsa-miR-1268a	2.1	5.76E-05	3.01E-03
20519525	hsa-miR-4707-5p	2.1	3.20E-04	1.06E-02
20525644	hsa-miR-6840-3p	2.1	2.56E-03	4.66E-02
20518629	hsa-miR-3940-5p	2.0	1.18E-06	1.96E-04
20526174	hsa-miR-7108-5p	2.0	2.80E-05	1.59E-03
20519580	hsa-miR-4734	2.0	1.61E-04	6.50E-03
20518913	hsa-miR-4516	2.0	1.04E-05	7.87E-04
20525396	hsa-miR-6724-5p	2.0	2.33E-05	1.40E-03
20526861	hsa-miR-7150	1.9	2.45E-03	4.54E-02
20525677	hsa-miR-6858-5p	1.9	1.25E-06	1.96E-04
20518782	hsa-miR-1268b	1.9	1.41E-03	3.15E-02
20519474	hsa-miR-4674	1.9	1.83E-03	3.72E-02
20517729	hsa-miR-4270	1.9	2.63E-03	4.75E-02
20514163	hsa-miR-2861	1.9	1.33E-04	5.79E-03
20523020	hsa-miR-6088	1.7	3.52E-04	1.11E-02
20519493	hsa-miR-4687-3p	1.6	2.80E-03	4.88E-02
20525699	hsa-miR-6869-5p	1.6	1.90E-03	3.81E-02
20525681	hsa-miR-6769b-5p	1.6	5.09E-04	1.41E-02
20525533	hsa-miR-6786-5p	1.6	1.58E-03	3.47E-02
20519507	hsa-miR-4695-5p	1.6	6.44E-04	1.68E-02
20529779	hsa-miR-8069	1.5	2.24E-03	4.27E-02
20504391	hsa-miR-638	1.5	1.62E-03	3.47E-02

20507742	hsa-miR-1469	1.5	1.53E-04	6.35E-03
20504586	hsa-miR-762	1.5	2.89E-04	1.01E-02
20519679	hsa-miR-4787-5p	1.4	2.86E-03	4.95E-02
20518905	hsa-miR-4508	1.4	2.75E-04	1.00E-02
20525416	hsa-miR-6727-5p	1.4	2.28E-03	4.30E-02
20525011	hsa-miR-6505-5p	1.4	7.44E-04	1.90E-02
20519639	hsa-miR-4765	1.3	2.70E-03	4.77E-02
20529765	hsa-miR-8055	-1.3	9.16E-04	2.17E-02
20501162	hsa-miR-200a-5p	-1.3	1.22E-03	2.79E-02
20500423	hsa-miR-30c-2-3p	-1.8	1.25E-03	2.83E-02
20500848	hsa-miR-320a	-1.8	2.11E-03	4.14E-02
20529139	hsa-miR-7847-3p	-1.9	2.71E-03	4.77E-02
20517680	hsa-miR-4298	-1.9	1.73E-03	3.55E-02
20506835	hsa-miR-1244	-2.0	8.22E-04	2.00E-02
20501157	hsa-miR-1244	-2.0	2.11E-03	4.14E-02
20509070	hsa-miR-320d	-2.0	7.76E-04	1.93E-02
20504563	hsa-miR-320c	-2.1	8.18E-04	2.00E-02
20500484	hsa-miR-221-3p	-2.1	2.13E-03	4.14E-02
20500196	hsa-miR-107	-2.2	1.22E-03	2.79E-02
20529785	hsa-miR-8075	-2.2	3.71E-04	1.15E-02
20500171	hsa-miR-92a-3p	-2.3	1.85E-03	3.74E-02
20500119	hsa-let-7d-5p	-2.6	1.70E-03	3.55E-02
20500761	hsa-miR-191-5p	-2.6	1.80E-04	7.02E-03
20500173	hsa-miR-93-5p	-2.8	3.88E-05	2.14E-03
20501280	hsa-miR-342-3p	-2.8	2.51E-03	4.61E-02
20500148	hsa-miR-24-3p	-2.9	1.86E-04	7.02E-03
20500162	hsa-miR-30a-5p	-2.9	2.71E-03	4.77E-02
20500130	hsa-miR-17-5p	-3.0	2.19E-03	4.21E-02
20500146	hsa-miR-23a-3p	-3.2	5.13E-04	1.41E-02
20500137	hsa-miR-19b-3p	-3.2	6.23E-04	1.67E-02
20500721	hsa-miR-23b-3p	-3.3	2.98E-04	1.01E-02
20500152	hsa-miR-26a-5p	-3.4	4.19E-04	1.24E-02
20500769	hsa-miR-126-3p	-3.8	7.63E-04	1.92E-02
20517816	hsa-miR-3609	-3.9	2.78E-05	1.59E-03
20500422	hsa-miR-30c-5p	-4.5	2.92E-04	1.01E-02
20500765	hsa-miR-125a-5p	-4.7	2.08E-04	7.72E-03
20500400	hsa-miR-199a-3p	-5.6	4.54E-04	1.30E-02
20500458	hsa-miR-199b-3p	-5.6	4.54E-04	1.30E-02
20500798	hsa-miR-195-5p	-5.6	3.23E-04	1.06E-02

Supplemental Table S3. Differentially expressed CNIT-specific genes related to oxidative phosphorylation.

Symbol	Entrez Gene Name	Affymetrix	Expr Fold Change
ATP5F1A	ATP synthase F1 subunit alpha	213738_s_at	-1.7
ATP5F1B	ATP synthase F1 subunit beta	201322_at	-2.4
ATP5F1C	ATP synthase F1 subunit gamma	208870_x_at	-1.6
ATP5F1D	ATP synthase F1 subunit delta	213041_s_at	-1.7
ATP5MC1	ATP synthase membrane subunit c locus 1	208972_s_at	-2
ATP5MC2	ATP synthase membrane subunit c locus 2	208764_s_at	-1.5
ATP5MC3	ATP synthase membrane subunit c locus 3	207508_at	-2.7
ATP5MF	ATP synthase membrane subunit f	202961_s_at	-1.6
ATP5MG	ATP synthase membrane subunit g	210453_x_at	-1.9
ATP5PD	ATP synthase peripheral stalk subunit d	210149_s_at	-2
ATP5PF	ATP synthase peripheral stalk subunit F6	202325_s_at	-1.6
ATP5PO	ATP synthase peripheral stalk subunit OSCP	200818_at	-1.7
COX17	cytochrome c oxidase copper chaperone COX17	203880_at	-1.8
COX4I1	cytochrome c oxidase subunit 4I1	202698_x_at	-1.5
COX5A	cytochrome c oxidase subunit 5A	203663_s_at	-1.8
COX5B	cytochrome c oxidase subunit 5B	213736_at	-1.9
COX6A1	cytochrome c oxidase subunit 6A1	200925_at	-1.5
COX6B1	cytochrome c oxidase subunit 6B1	201441_at	-1.8
COX6C	cytochrome c oxidase subunit 6C	201754_at	-2.2
COX7A2	cytochrome c oxidase subunit 7A2	201597_at	-1.8
COX7B	cytochrome c oxidase subunit 7B	202110_at	-2.2
COX7C	cytochrome c oxidase subunit 7C	213846_at	-2.9
COX8A	cytochrome c oxidase subunit 8A	201119_s_at	-2.7
CYB5A	cytochrome b5 type A	215726_s_at	-1.9
CYC1	cytochrome c1	201066_at	-2
NDUFA1	NADH:ubiquinone oxidoreductase subunit A1	202298_at	-1.8
NDUFA2	NADH:ubiquinone oxidoreductase subunit A2	209224_s_at	-2.2
NDUFA4	NDUFA4 mitochondrial complex associated	217773_s_at	-2.5
NDUFA7	NADH:ubiquinone oxidoreductase subunit A7	202785_at	-1.7
NDUFA8	NADH:ubiquinone oxidoreductase subunit A8	218160_at	-1.6
NDUFA9	NADH:ubiquinone oxidoreductase subunit A9	208969_at	-1.6
NDUFA13	NADH:ubiquinone oxidoreductase subunit A13	220864_s_at	-1.5
NDUFAB1	NADH:ubiquinone oxidoreductase subunit AB1	202077_at	-1.7
NDUFB2	NADH:ubiquinone oxidoreductase subunit B2	218200_s_at	-2.9
NDUFB3	NADH:ubiquinone oxidoreductase subunit B3	203371_s_at	-1.5
NDUFB4	NADH:ubiquinone oxidoreductase subunit B4	218226_s_at	-1.8
NDUFB6	NADH:ubiquinone oxidoreductase subunit B6	203613_s_at	-2.1
NDUFB8	NADH:ubiquinone oxidoreductase subunit B8	201227_s_at	-2
NDUFS1	NADH:ubiquinone oxidoreductase core subunit S1	203039_s_at	-1.8
NDUFS2	NADH:ubiquinone oxidoreductase core subunit S2	201966_at	-1.5

NDUFS3	NADH:ubiquinone oxidoreductase core subunit S3	201740_at	-1.7
NDUFS4	NADH:ubiquinone oxidoreductase subunit S4	209303_at	-1.8
NDUFS7	NADH:ubiquinone oxidoreductase core subunit S7	211752_s_at	-1.8
NDUFS8	NADH:ubiquinone oxidoreductase core subunit S8	203189_s_at	-1.9
NDUFV2	NADH:ubiquinone oxidoreductase core subunit V2	202941_at	-1.5
SDHB	succinate dehydrogenase complex iron sulfur subunit B	202675_at	-1.8
SDHC	succinate dehydrogenase complex subunit C	216591_s_at	-2.3
SURF1	SURF1 cytochrome c oxidase assembly factor	204295_at	-1.6
UQCR10	ubiquinol-cytochrome c reductase, complex III subunit X	218190_s_at	-1.9
UQCR11	ubiquinol-cytochrome c reductase, complex III subunit XI	202090_s_at	-2.2
UQCRC1	ubiquinol-cytochrome c reductase core protein 1	201903_at	-1.9
UQCRC2	ubiquinol-cytochrome c reductase core protein 2	200883_at	-1.6
UQCRFS1	ubiquinol-cytochrome c reductase, Rieske iron-sulfur polypeptide 1	208909_at	-2
UQCRQ	ubiquinol-cytochrome c reductase complex III subunit VII	201568_at	-2.1
